# New Antioxidant Triphenol-Derived Hydrazide-Hydrazone Thiazole: Formation and Analysis of Inclusion Complex with β-CD Using Experimental and Computational Approaches

**DOI:** 10.3390/molecules30081842

**Published:** 2025-04-19

**Authors:** Adrian Pîrnău, Mihaela Mic, Călin G. Floare, Ovidiu Oniga, Smaranda Dafina Oniga, Ovidiu Crișan, Laurian Vlase, Gabriel Marc

**Affiliations:** 1 National Institute for Research and Development of Isotopic and Molecular Technologies, 67-103 Donat, 400293 Cluj-Napoca, Romania; adrian.pirnau@itim-cj.ro (A.P.); calin.floare@itim-cj.ro (C.G.F.); 2 Department of Pharmaceutical Chemistry, Faculty of Pharmacy, “Iuliu Hațieganu” University of Medicine and Pharmacy, 41 Victor Babeș Street, 400012 Cluj-Napoca, Romania; ooniga@umfcluj.ro; 3 Department of Therapeutic Chemistry, “Iuliu Hațieganu” University of Medicine and Pharmacy, 12 Ion Creangă Street, 400010 Cluj-Napoca, Romania; smaranda.oniga@umfcluj.ro; 4 Department of Organic Chemistry, Faculty of Pharmacy, “Iuliu Hațieganu” University of Medicine and Pharmacy, 41 Victor Babeș Street, 400012 Cluj-Napoca, Romania; ocrisan@umfcluj.ro (O.C.); marc.gabriel@umfcluj.ro (G.M.); 5 Department of Pharmaceutical Technology and Biopharmaceutics, “Iuliu Hațieganu” University of Medicine and Pharmacy, 41 Victor Babeș Street, 400012 Cluj-Napoca, Romania; laurian.vlase@umfcluj.ro

**Keywords:** polyphenol antioxidant activity, β-cyclodextrin, inclusion complex

## Abstract

A new water-soluble not-colored antioxidant (Z)-N′-(4-(3,4-dihydroxyphenyl)-3-ethylthiazol-2(3H)-ylidene)-4-hydroxybenzohydrazide hydrochloride (DHTH) was obtained and characterized. The interaction between DHTH and β-CD was studied by experimental thermodynamic methods such as isothermal titration calorimetry (ITC) and ^1^H NMR spectroscopy and confirmed by in silico calculations. Thermodynamic data indicated that the inclusion process is driven by enthalpy, predominantly as a result of the guest–host hydrophobic interactions. ^1^H NMR measurements were applied to study the interaction with β-CD by changing the studied compound concentration in the solution. UV-vis titration and in vitro antiradical assay were performed, to study the antioxidant activity of DHTH, free and included in β-CD. A molecular docking study added supplementary insight to the experimental analyses regarding the binding conformation of the new polyphenolic compound to β-CD.

## 1. Introduction

Oxidative stress arises when the generation of reactive oxygen species exceeds the biological system’s capacity to neutralize them or repair the damage they cause to lipids, proteins, and DNA. It is a natural consequence of living in an aerobic environment but can be exacerbated by conditions such as diabetes, neurodegenerative disorders, cancer, and vascular or immune diseases. Preventing oxidative stress is a key strategy in reducing complications associated with these conditions. Protection against free radicals can be enhanced by increasing protective factors, which may come from various sources, such as diet or pharmaceutical interventions, including vitamins E, A, and C, obtained from natural or synthetic sources [1,2,3,4]. Phenolic compounds are widely recognized for their strong antioxidant properties. In recent years, there has been significant interest in discovering new phenolic compounds with antioxidant potential, whether derived from plants or synthesized chemically [5,6,7,8].

**Figure 1:**
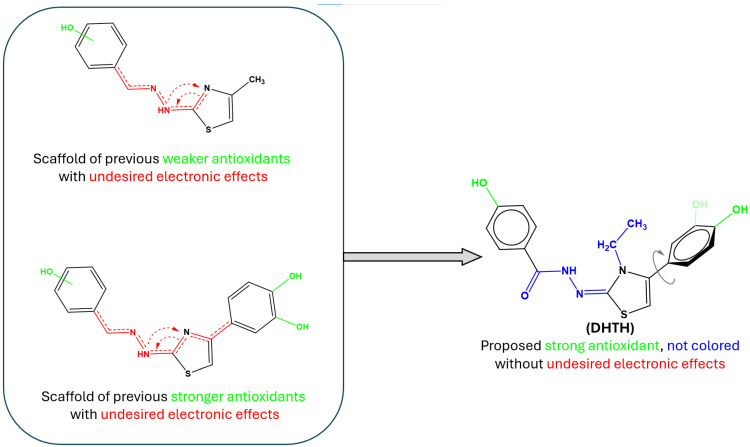
The rationale behind the synthesis of the new compound DHTH, based on our group previous works [8,9,10].

The DHTH ((Z)-N′-(4-(3,4-dihydroxyphenyl)-3-ethylthiazol-2(3H)-ylidene)-4-hydroxybenzohydrazide hydrochloride) design was based on scaffold hopping, based on our group’s previous experience in the field of polyphenol-derived thiazoles with antioxidant activity (Figure 1). In the compounds previously obtained by our group, the drawback of being colored yellow orange was constant for all compounds [9], which may be unsuitable for antioxidant compounds with a role in industry of preservation and protection from the oxidizing action of atmospheric oxygen in dietary supplements, etc. Being colored, an antioxidant may absorb the visible light, which may lead to photodegradation with consequent reduction in the antioxidant effectiveness over time and it may also promote photooxidation, paradoxically increasing oxidative stress rather than preventing it [10,11,12]. This yellow–orange color was identified in compounds that have the antioxidant catechol moiety in the position 4 of the thiazole nucleus or those that have the hydrazone group in their structure. However, the catechol at position 4 of the thiazole ring was kept, because it has shown very good antioxidant activity in our previous research and introduced an ethyl substituent on the nitrogen atom of the thiazole ring to induce a rotational change of the covalent bond between thiazole and catechol. This structural modification with an electron-inductive effect aimed at multicenter electron conjugation perturbation, which may disrupt conjugation and slightly shift UV-Vis absorption of the proposed compound. The proposed alkyl substitution of the endocyclic nitrogen with an electron donating group targeted the disruption of the tautomerism between 2-amino thiazole and 2-imino thiazole, which can lead to an unwanted extended conjugation of electrons over the molecules leading to absorption in the visible spectra [13,14]. More than that, the insertion of the respective moiety on the nitrogen atom would rotate the catechol ring out of the thiazole plane, perturbing the conjugation between the catechol and thiazole, a hypothesis confirmed in the DFT study.

The hydrazone linker, a chromophore found in most of our previous compounds, was replaced in the present compound with a hydrazide, with no absorption in the visible spectrum, a well-known moiety used as a ligand for transition metals ions chelator [15,16,17,18]. The respective ions can undergo catalyzation by Fenton or Haber–Weiss reactions in biological environments leading to an overproduction of reactive oxygen species and depletion of antioxidant protection resources [19,20,21]. By insertion of transition metals chelator moieties (metallophores), the antioxidant activity of the compounds can be supplemented in a complementary way, by sequestering the respective free ions involved in radical species generation [22,23,24,25]. More than that, the catechol group from DHTH is known to be chelator of some transition ion metals such as copper or iron [26,27].

The inclusion of compounds in cyclodextrins (CDs) has become a widely used strategy to enhance their properties, including solubility, stability, reduced tissue irritation, and masking of odor or taste, thanks to the amphiphilic properties of CDs; the internal cavity is hydrophobic and the outside is hydrophilic. Cyclodextrins are, nowadays, produced in large quantities as they have many uses in food, pharmaceutical, and chemical industries, or in agriculture [28,29,30,31]. Some studies suggested that after complexation with βCD, the antioxidant capacity increased significantly, while in other situations, no improvement was observed in the complex’s activity. Therefore, it is important to understand the interaction mechanism and the characterization of the formation of the inclusion complex [32,33,34,35,36,37].

The supramolecular inclusion compounds of cyclodextrins are very diverse and have been intensively studied. Cyclodextrins are evaluated to be safe by the FDA [38] and are ingredients in more than 30 different approved medicines [39]. The most frequently used natural cyclodextrin is β-cyclodextrin (β-CD), a macrocyclic oligosaccharide, comprising seven α-D-glucopyranose units produced by enzymatic conversion of natural starch, an important polysaccharide from plants [40].

The present study was focused on the development of DHTH, a non-colored polyphenolic antioxidant, based on the scaffold hoping method, and on the experimental nuclear magnetic resonance (NMR) and isothermal titration calorimetry (ITC) analysis of its complex with β-CD [41]. Among the natural cyclodextrins α, β and γ, β cyclodextrins have the most appropriate internal diameter for the incorporation of molecules that have a benzene or thiazole ring in their structure. 

The approach to include antioxidant compounds highly susceptible to degradation through oxidation or photodegradation such as polyphenols in CDs has been extensively documented in the literature, with CDs being considered as “secondary antioxidants”, due to enhancing the stability of the included antioxidant molecule [35,36,42,43,44,45,46,47,48,49].

In addition to the experimental investigations, the most probable molecular conformations of the supramolecular inclusion compound of DHTH:β-CD were also theoretically investigated using molecular docking.

## 2. Results and Discussion

### 2.1. Chemical Synthesis

The intermediate compound N-ethyl-2-(4-hydroxybenzoyl)hydrazine-1-carbothioamide (**3**) was obtained from the condensation between 4-hydroxybenzohydrazide (**1**) and ethyl isothiocyanate (**2**) in ethanol. Obtention of the desired compound **5** was made using the intermediate carbothioamide (**3**) and 4-(chloroacetyl)catechol (**4**) in boiling acetone. After following the steps described from the synthesis protocol, the intermediate compound **3** and the desired final compound **5** were obtained and spectral data were recorded to confirm their successful obtention (Figures S1–S5).

*N-ethyl-2-(4-hydroxybenzoyl)hydrazine-1-carbothioamide* (**3**): white solid; 202–203 °C (lit. 204–205 °C [50]); yield = 73%; FT IR (KBr) ν_max_ cm^−1^: 3553, 3474, 3414, 3352, 3337, 3200, 2978, 2931, 2858, 1636 (C=O), 1607, 1591, 1553, 1546, 1490, 1469, 1451, 1432, 1380, 1312, 1292, 1283, 1232, 1181, 1126, 1089, 1070, 1050, 894, 851, 805, 775; MS: *m*/*z* = 238.0 (M-1); ^1^H-NMR (DMSO-d6, 500 MHz) δ: 10.062–10.041 (br, 2H), 9.131 (br, 1H), 8.007 (br, 1H), 7.780 (d, 2H, Ar, *J* = 8.50 Hz), 6.812 (d, 2H, Ar, *J* = 9.0 Hz), 3.457 (q, 2H, -CH_2_-), 1.050 (t, 3H, -CH_3_, *J* = 7 Hz); ^13^C-NMR (DMSO-d6, 125 MHz) δ: 166.1 (C=O), 161.2 (ArC-OH), 130.3 (Ar), 123.6 (Ar), 115.2 (Ar), 38.9 (-CH_2_-), 15.0 (-CH_3_).

*N′-(4-(3,4-dihydroxyphenyl)-3-ethylthiazol-2(3H)-ylidene)-4-hydroxybenzohydrazide hydrochloride* (**5**): white solid; 272–273 °C; yield = 61%; IR cm^−1^: 3293 (νO-H), 3193 (νO-H), 3122 (νN-H), 2992, 2974, 2934 (νC-H), 2891, 2842 (νC-H), 2806, 1695 (νC=O), 1687 (νC=N), 1608 (νC=C), 1589, 1518, 1471, 1435, 1379, 1355, 1289, 1266, 1242, 1221, 1175, 1115, 1050, 962, 897, 849, 821, 788, 764, 749, 642, 625; MS: *m*/*z* = 370.1 (M-1); ^1^H-NMR (D_2_O, 500 MHz) δ: 7.79 (d, 2H, Ar, *J* = 8.79 Hz), 6.98 (d, 1H, Ar, *J* = 8.22 Hz), 6.97 (d, 2H, Ar, *J* = 8.79 Hz), 6.96 (d, 1H, Ar, *J* = 2.20 Hz), 6.90 (dd, 1H, Ar, *J*_1_ = 8.22 Hz and *J*_2_ = 2.20 Hz), 6.88 (s, 1H, Th), 4.00 (q, 2H, -CH_2_-, *J* = 7.33 Hz), 1.24 (t, 3H, -CH_3_, *J* = 7.33 Hz); ^13^C-NMR (D_2_O, 125 MHz) δ: 172.0 (C=O), 160.7 (ArC-OH), 146.4 (ArC-OH), 144.2 (Th), 143.5 (ArC-OH), 130.2 (Ar), 123.1 (Ar), 121.8 (Ar), 120.0 (Ar), 117.6 (Ar), 116.2 (Ar), 115.8 (Ar), 106.4 (Th).

The infrared spectra of the intermediate thiosemicarbazide **3** and final compound **5** (DHTH) are consistent with the expected data. In the 3200–3500 cm^−1^ zone of the intermediate compound **3,** multiple broad signals were identified, due to the stretching of the OH and NH bonds, confirmed by the presence of the multiple bending signals of the respective groups, but difficult to trace. Two significant changes were identified in the infrared spectrum, after successful conversion of the compound **3** in compound **5**. First, in the 3100–3400 cm^−1^ zone, some large overlapping signals attributed to the stretching of the three OH groups in compound **5** appeared, when compared to the intermediate compound **3**. Second, the stretching of the C=O bond shifted from 1636 cm^−1^ in compound **3** to 1695 cm^−1^ in compound **5**, due to the cyclization of the benzoylated thiosemicarbazide to the hydrazide-hydrazone-like compound **5**.

In the mass spectra of compounds **3** and **5** was identified the molecular peak corresponding to the anions of the respective compounds (M-1).

In the NMR spectrum of compound **5**, all protons were identified, with the expected chemical shift, with the expected integrals and multiplicity. Detailed comments and interpretation are presented in the following section.

In the ^13^C NMR spectrum of compound 5, the signals could be attributed to the carbon atoms in the structure and distinctive signals could be identified, such as the C=O at 172.0 ppm, the three carbon atoms with phenol groups at 160.7 ppm, 146.4 ppm, and 143.5 ppm, and the aliphatic carbons at 43.2 ppm and 30.2 ppm.

### 2.2. Determination of the Stoichiometry by NMR

**Figure 2:**
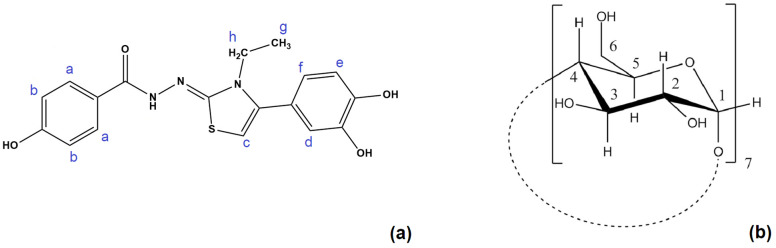
Structure and proton numbering of (**a**) DHTH and (**b**) β-CD.

**Figure 3:**
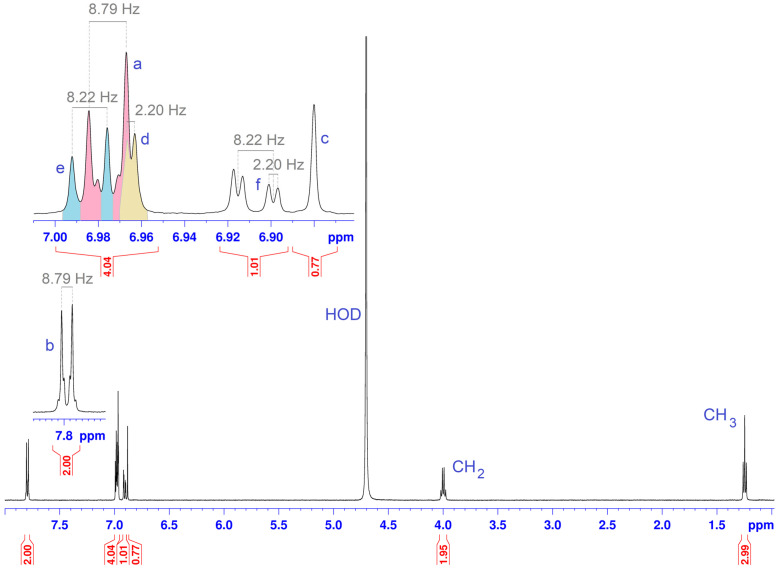
^1^H NMR spectrum of DHTH in D_2_O-water suppression.

**Figure 4:**
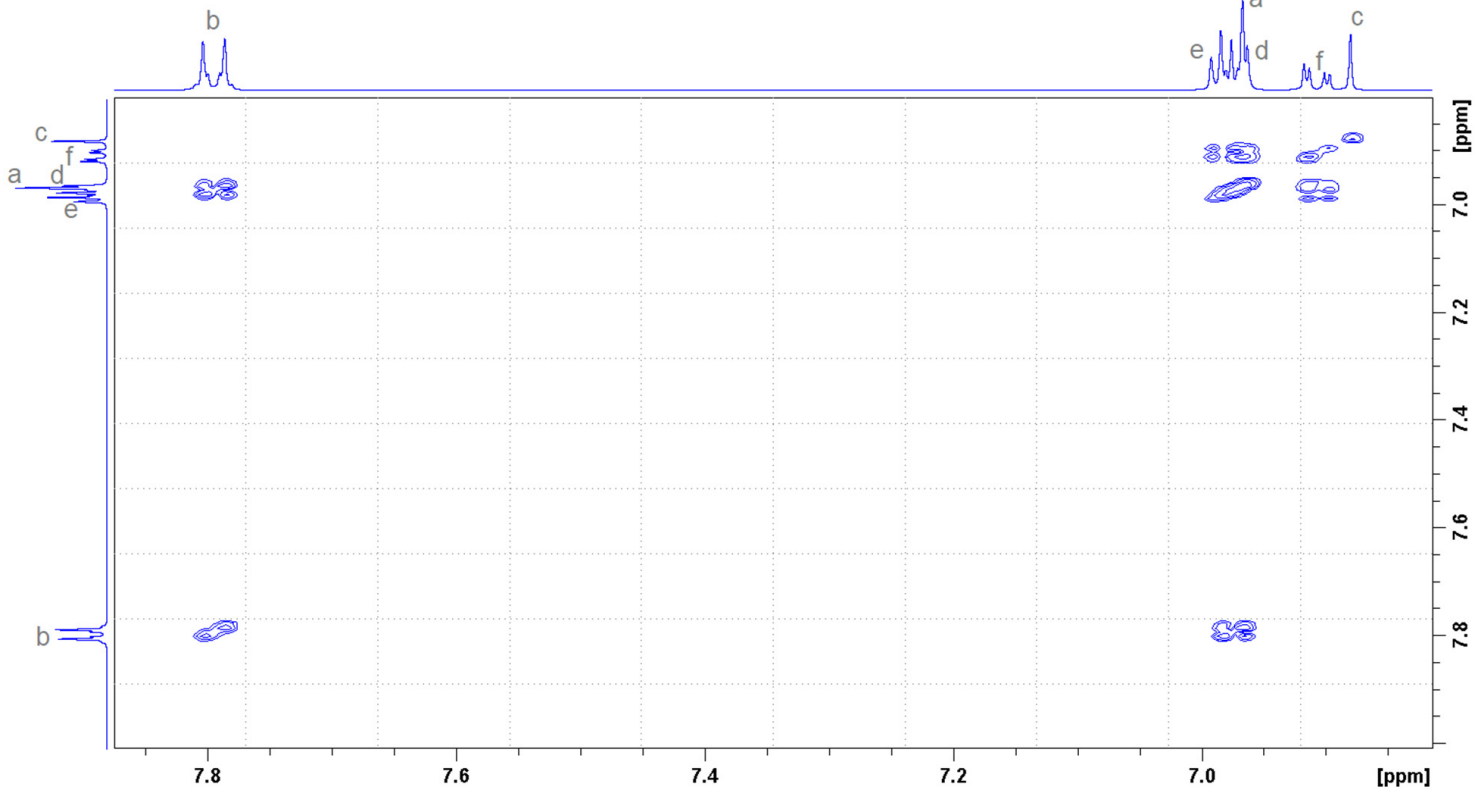
^1^H-^1^H COSY NMR spectrum of DHTH in D_2_O, the aromatic area.

Figure 2 presents the molecules and the atoms numbering of DHTH and β-CD. Figure 3 shows the ^1^H NMR spectrum for the DHTH, with the assignments based on the 2D spectrum ^1^H-^1^H COSY NMR (Figure 4). Therefore, the complete interpretation of the ^1^H NMR spectrum of DHTH in free state is summarized in Table 1, presenting the chemical shifts of the protons, as well as the identification of the multiplicity with the related coupling constants. In the aromatic spectral range, several types of signals can be identified that come from pairs of protons, with signals colored differently to highlight exactly the state of multiplicity with associate coupling constants, and to allow us to compare these signals with the following pairs: a with b; f with e and d. Regarding the signals of the protons coming from the amine and OH groups, they are superimposed on the signal of the protons from HOD.

**Figure 5:**
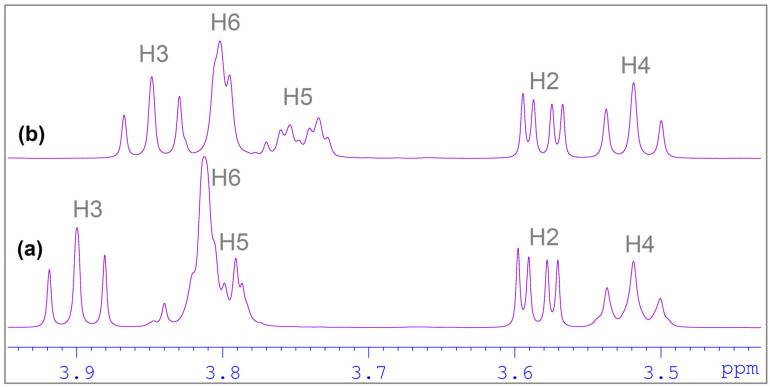
^1^H NMR spectra between 3.4 ppm and 4.0 ppm, at different ratio concentrations: (**a**) 4 mM β-CD (host) and (**b**) 2 mM β-CD (host alone) and 2 mM DHTH (guest).

**Figure 6:**
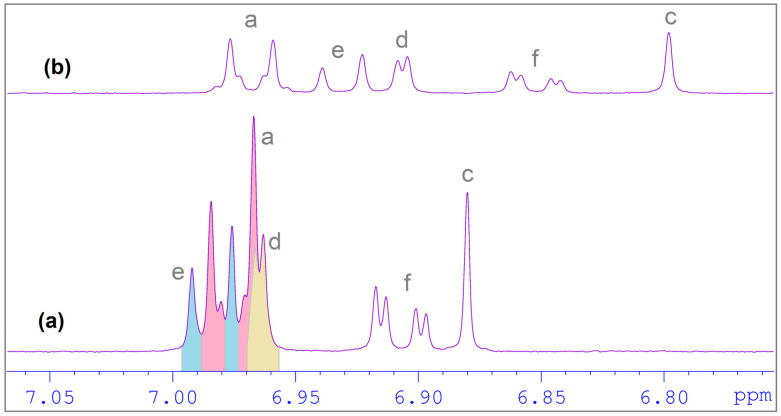
^1^H NMR spectra in the aromatic zone (6.7–7.1 ppm), at different ratio concentrations: (**a**) 4 mM DHTH (host alone) and (**b**) 2 mM β-CD (host) and 2 mM DHTH (guest).

To confirm the formation of the DHTH:β-CD complex, ^1^H NMR spectroscopy was used. ^1^H NMR is a technique which provides the most evidence of inclusion of a guest molecule into the hydrophobic β-CD cavity in solution [51,52]. The inclusion of DHTH in β-CD is shown by the change in the chemical shift of some guest DHTH and host (β-CD) protons (in the mixture), in comparison with the chemical shifts of the same protons in the free components. The method relies on the change in chemical shifts (Δδ) of the protons involved in the interaction between the studied molecules (DHTH and β-CD). The comparison is made relative to the signals in the free state (without the other molecule). Partial ^1^H NMR spectra of components and DHTH:β-CD equimolar mixture is depicted in Figure 5 and Figure 6. No new peaks appeared in the spectra recorded for the studied complexes, suggesting the studied inclusion is dynamic.

**Figure 7:**
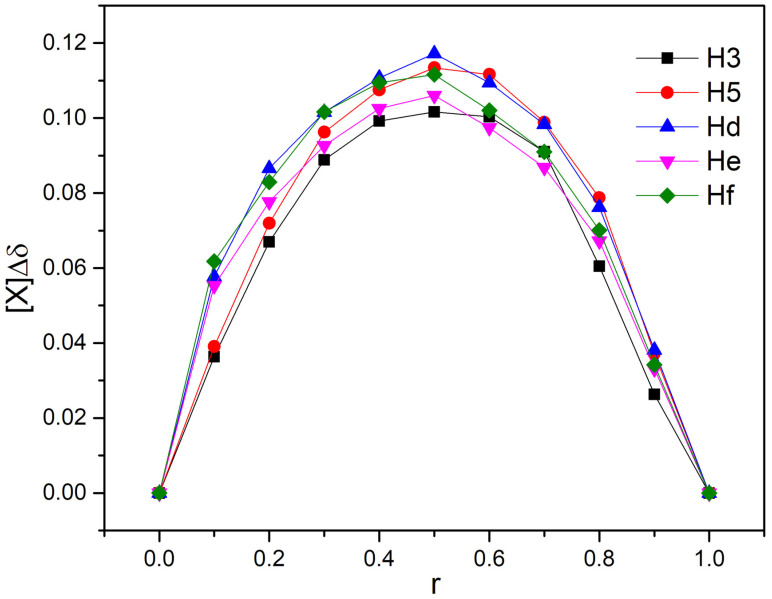
Concentration change plot for H3 and H5 protons from β-CD, and Hd, He and Hf protons from DHTH, where [X] = [DHTH] or [β-CD].

The ^1^H NMR spectra of DHTH when mixed with β-CD showed changes of the chemical shifts (Δδ) of some protons when the two compounds were mixed, when compared to free DHTH. The signals of protons from the inside cavity of the β-CD (H3 and H5) experienced a chemical shift (Δδ) in the presence of DHTH, which confirms the interaction between the DHTH and the inside of the β-CD, which served as evidence the formation of the DHTH:β-CD complex. By applying the continuous variation method, the stoichiometry of the DHTH:β-CD complexation process was found, which follows Δδ dependent on the concentrations [52]. Since graphs (Figure 7) have symmetry, with a 0.5 maximal value for r, it can be assumed that the inclusion has an equimolar stoichiometry. This can be observed both for the protons from DHTH and for the protons inside β-CD (H3 and H5).

**Figure 8:**
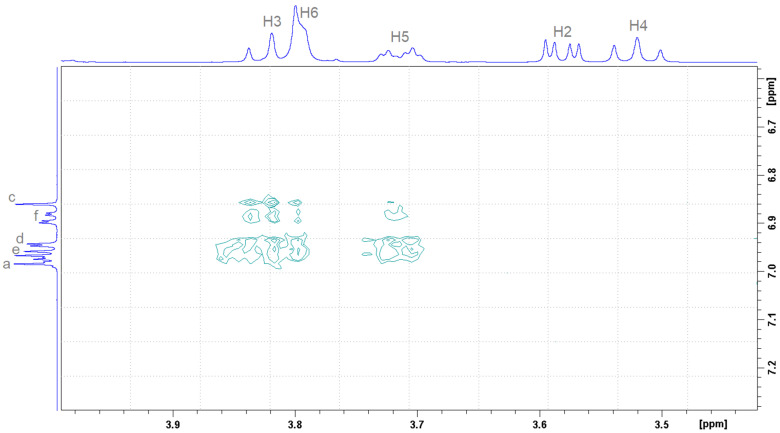
Two-dimensional ROESY NMR spectrum of DHTH: β-CD complex in equimolecular ratio (2 mM each for host and guest).

Two-dimensional (^1^H-^1^H) ROESY can highlight non-covalent interactions between protons through space between different molecules. Figure 8 depicts the interactions between aromatic (Ha, Hd, He, Hf) and thiazole (Hc) protons belonging to DHTH molecule with protons inside β-CD (H3, H5), proving the inclusion of DHTH inside β-CD cavity.

To determine the extent of the intermolecular binding between DHTH and β-CD, the association constant was evaluated. The association constant *K* for a 1:1 complex can be determined according to the following equation [17]:

(1) Δδi,j=Δδcj2XC+1K−C+1K2−4AiBi12

where *i* represents the sample number and *j* the traced proton. If the studied proton belongs to the guest or host molecule, *X* = *A* or *B*, respectively. Δδ*_c_*^(*i*)^ represents the chemical shift difference between the free component and the pure inclusion complex. Equation (1) involves no approximations and correlates the total concentrations of the guest and host molecules with the observed change in Δδ:

(2) $$\Delta \delta^{\left(i,j\right)}=\delta_{free}^{\left(i,j\right)}-\delta_{obs}^{\left(i,j\right)}$$

Using CONSTEQ software [52], the calculation of K_a_ was performed, using an iteration following specific algorithms to fit the experimental values of Δδ(*i*,*j*) to the appropriate equation, the mathematical description being presented in a previous publication [51,52]. Thus, an association constant K_a_ = 81.97 M^−1^ was obtained, with a very good fit to the experimental points with a correlation factor R = 0.993.

### 2.3. Isothermal Titration Calorimetry

**Figure 9:**
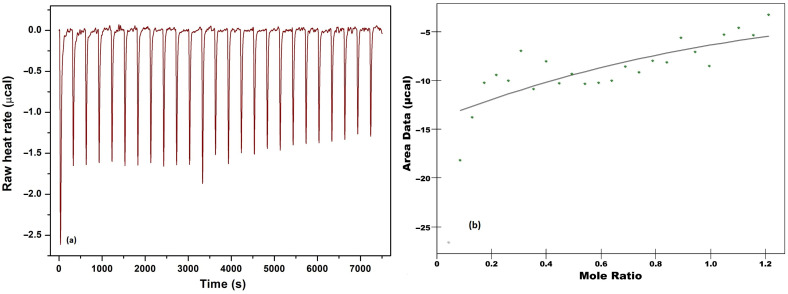
Isothermal titration calorimetric profile for the interaction of DHTH to β-CD at 25 °C. (**a**) The data obtained during 25 consecutive injections of 10 μL of DHTH (3 mM) in β-CD (0.75 mM). (**b**) The incremental heat/mol of added DHTH dependant of molar [DHTH]/[β-CD] ratio.

To understand the nature of the interaction between DHTH and β-CD, ITC experiments were performed, which allow the simultaneous determination of the binding enthalpy, the binding constant and the stoichiometry in a single experiment. A representative calorimetric titration profile of the binding of DHTH to β-CD is shown in Figure 9. Exothermic heat flows, which were released after successive injections of 10 μL aliquots of DHTH into a sample cell containing β-CD solution, were integrated and expressed as a function of the molar ratio between the two compounds.

The complex formation constant (K_a_ = 1.096 × 10^3^ M^−1^) indicates that a stable inclusion complex is formed between DHTH and β-CD. The exothermic nature of the host–guest interaction is attributed to the negative enthalpy change (ΔH = −1.013 kcal/mol), which results from the association between the small guest molecule and the hydrophobic cavity of β-CD in aqueous solution [53]. The entropic effect of the complexation process (ΔS = 10.51 cal/mol·K) contributes to the negative Gibbs free energy. A negative value of Gibbs free energy change (ΔG = −4.144 Kcal/mol) indicates that the binding of DHTH to β-CD is a spontaneous process. The change in total Gibbs free energy is the consequence of an integrated effect involving both the entropic and enthalpy contributions. The disruption of the ordered aqueous microenvironment around the hydrophobic regions of the guest molecule upon its binding to β-CD is the driving process in the changes in enthropy. The hydrophobic interaction releases water molecules from the inside of β-CD, while the increase in the entropy is the driving process of the studied interaction. Calorimetric measurements of the interaction of DHTH with β-CD have shown that the stoichiometry of the host–guest complex is 1:1, which is in agreement with the results obtained from NMR analysis.

### 2.4. Molecular Docking

**Figure 10:**
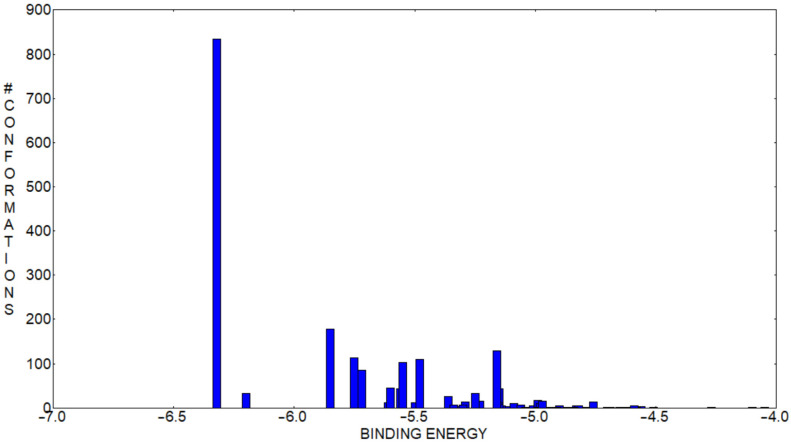
Histogram of the clusters with predicted conformations of DHTH inside of β-CD.

To understand the most probable inclusion conformation of DHTH inside β-CD, a molecular docking simulation was performed. Using a root mean square deviation of 2 Å, out of the 2000 docking runs, 57 conformational clusters were obtained. The binding affinity distribution from the identified clusters where the intensity represents the number of conformations belonging to each cluster and the position on the abscissa are determined by the leading binding energy member and are represented in Figure 10. The conformational movement of DHTH inside the cavity of β-CD is hindered as pointed out by the relatively reduced number of clusters. This is, however, a typical case for the inclusion compounds of β-CD interacting with relatively small guest molecules.

**Figure 11:**
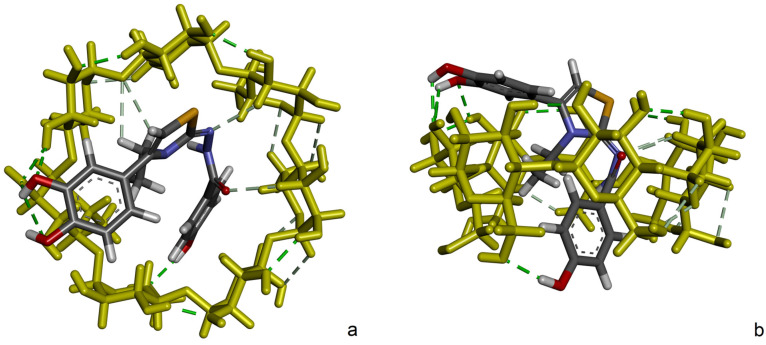
The leading binding conformation of DHTH:β-CD complex (ΔG = −6.32 kcal/mol). The inter- and intra-molecular hydrogen bonds are also represented using green dashed lines. (**a**) Secondary rim view and (**b**) side view.

Figure 11 shows the conformation of DHTH with the best affinity for β-CD (ΔG = −6.32 kcal/mol), rendered in two graphical representations. Out of the 2000 conformations generated, this one appears in 834 of them, and it is by far the most stable and favorable identified conformation. Additionally, the mean binding energy ΔG = −5.81 kcal/mol in the 2Å RMSD cluster of conformations is only ~0.5 kcal/mol higher than the presented conformation. DHTH crosses through the β-CD from one side to another with benzohydrazide and ethylthiazole moieties inside the cavity and the catechol moiety outside of the β-CD core, closer to the larger secondary β-CD rim.

In this most favorable conformation, the highest number of protons of DHTH interact with those of β-CD. With this, as mentioned, dihydroxyphenyl protons (Hd, He, Hf) which have the highest chemical shift variations are located close to the secondary rim.

**Figure 12:**
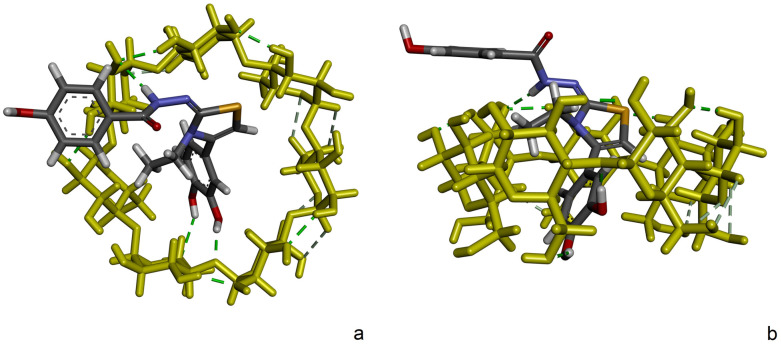
The alternative binding conformation of DHTH:β-CD inclusion compound with a ΔG = −5.23 kcal/mol. (**a**) Secondary rim view and (**b**) side view.

From ROESY data, it can also be seen that the 4-hydroxybenzohydrazide (Ha) equivalent protons and thiazole (Hc) protons had visible cross peaks with β-CD (H3, H5) protons. With this in mind, and after reviewing all identified binding conformations distributed in clusters, another binding conformation (ΔG = −5.23 kcal/mol) from the second cluster attracts attention, where dihydroxyphenyl and ethylthiazole moieties are located inside the cavity of β-CD. The respective binding pose is depicted in Figure 12.

### 2.5. UV Spectroscopy

**Figure 13:**
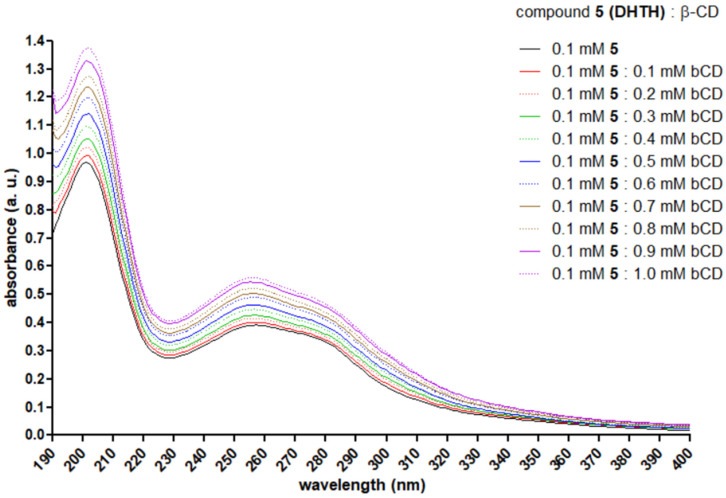
The UV spectrum recorded for DHTH between 190 and 400 nm in water, with increasing concentrations of β-CD.

The UV spectra recorded for the solutions of DHTH and increasing concentrations of β-CD are presented in Figure 13. In the water solution of DHTH, a significant peak was observed at 201 nm (C=O n → π*), an intermediate one at 255 (benzene π → π*) nm and a lower one at 275 nm (benzene π → π*), depicted with black. As long as the concentration of β-CD was increased, a hyperchromic effect was observed, in agreement with literature reports presenting compounds included in CDs [54,55,56]. No bathochromic or hypsochromic shifts were observed in the recorded spectra.

### 2.6. Antioxidant Activity Evaluation

**Figure 14:**
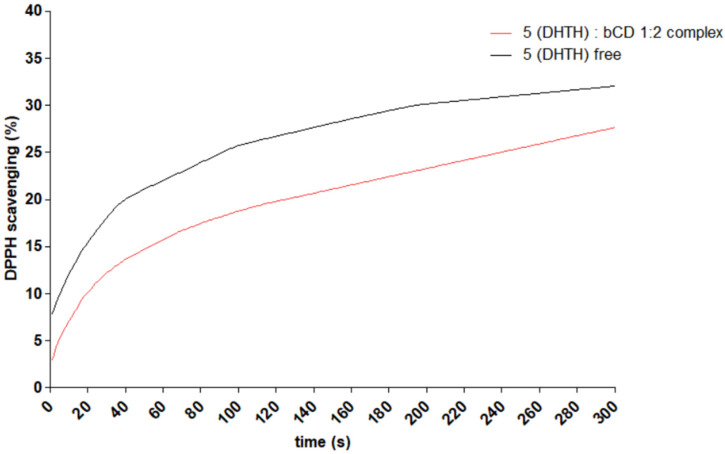
Comparative DPPH^•^ scavenging assay of compound **5** (DHTH) free and complexed with β-CD.

For evaluation of the difference in antioxidant behavior of free DHTH and that complexed with β-CD, the DPPH scavenging assay was performed, using the same concentration of DHTH in both samples. The absorbance of the samples was measured every 1 s for 300 s. The change in absorbance of samples in time is depicted in Figure 14. A significant difference between the evolution of the absorbance of the samples was identified. Free DHTH neutralizes DPPH radicals faster, when compared to the corresponding β-CD complex. The effect is faster in the first seconds of the assay, after which the effect appears slower.

## 3. Materials and Methods

### 3.1. Chemical Synthesis

The consumables and chemicals utilized in this study were of suitable grade purity produced by Sigma-Aldrich (Schnelldorf Deutschland, Germany), Carl Roth (Karlsruhe, Germany), Merck (Merck KGaA, Darmstadt, Germany), Alfa-Aesar (Thermo Scientific, Waltham, MA, USA), Eurisotop (Essonne, France) and sourced from the local suppliers Nordic Chemicals, Redox Chemicals, Solantis, Global Step, Bio-Aqua. Melting points of the solid compound powders were measured using a BUCHI M-560 device. The purity of compounds was preliminarily confirmed by TLC on silica gel 60 F254 plastic sheets (Merck, Darmstadt, Germany) using heptane:ethyl acetate mixtures in various ratios as mobile phases. which was later confirmed by RP-HPLC using an Agilent 1100 system, associated with an Agilent Ion Trap SL (Agilent Technologies, Santa Clara, CA, USA), in negative ionization mode. The infrared (IR) spectra of the compounds **3** and **5** compressed in KBr pills were recorded on a FT/IR 6100 apparatus (Jasco, Cremella, Italy), with 16 scans and 2.0 cm^−1^ resolution.

**Figure 15:**
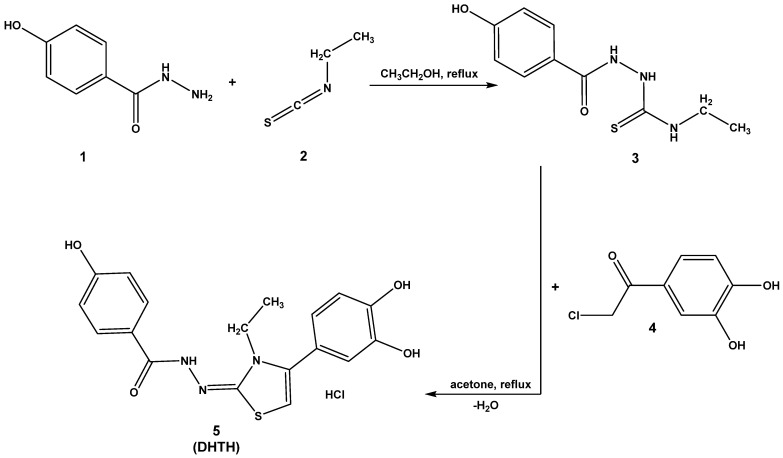
The synthesis pathway for obtention of DHTH (**5**).

The route followed for the obtention of the desired DHTH (**5**) via the intermediate thiosemicarbazide **3** is depicted in Figure 15.

For the obtention of the intermediate compound **3**, 0.76 g (5 mmol) of 4-hydroxybenzohydrazide (**1**) was dissolved in ethyl alcohol, and 0.44 g (5.1 mmol) of ethyl isothiocyanate was added. The resulting mixture was refluxed under condenser until completion of the reaction (approx. 60 min) [57]. The mixture was cooled to room temperature and left overnight to precipitate. The resulting solid was filtered using vacuum and washed with diethyl ether.

For the obtention of the final product DHTH (**5**), 0.47 g (2 mmol) of intermediate thiosemicarbazide **3** was dissolved in boiling acetone and 0.37 g (2 mmol) of 4-(chloroacetyl)catechol (**4**) was added. The reaction mixture was heated for approximately 2 h, in the dark and inert atmosphere. After reaction completion, the reaction mixture was cooled at the room temperature, mixed with diethyl ether and left overnight to precipitate. The desired product was filtered using vacuum and crystallized and washed with diethyl ether.

### 3.2. NMR Spectroscopy

NMR measurements were conducted using a Bruker AVANCE III 500 spectrometer (Karlsruhe, Germany) in the liquid state at 298 K, in D_2_O, with chemical shifts referenced to 3-(trimethylsilyl)-1-propanesulfonic acid salt (TMSP), which corresponds to 0 ppm in all spectra.

To show evidence of the DHTH:β-CD complex by NMR spectroscopy, 4 mM solutions in deuterated water of host and guest molecules were prepared, which were mixed in different concentration ratios resulting in 11 binary mixtures, in a complete range (0 < r < 1) of the ratio r = [X]/([H] + [G]), where X = H or G and [H] and [G] are the concentrations of the host (β-CD) and guest (DHTH), respectively, with 4 mM constant total concentration for each sample.

Thus, for the respective samples, the ^1^H NMR experiments were performed, where 128 scans were made, with 65 K points accumulated over a 5 kHz domain equivalent to 10 ppm, with a 4 s relaxation delay (D1) and an excitation pulse of 10.1 μs. The 2D NMR ROESY measurements were performed on a sample comprised by an equimolar mixture of DHTH and β-CD in. The acquisition parameters were the ones previously reported by our group, but with 16 scans [58]. The 2D ^1^H-^1^H COSY NMR measurement was made using 16 scans, consisting of a matrix of 2048 × 1024 data points and a spectral width of 5 kHz. ^13^C NMR measurement was performed, where 4096 scans were made with a 65 K points over 28.8 kHz range equivalent to 229 ppm, with 3 s relaxation delay (D1) and an excitation pulse of 8 μs.

The final output of the NMR measurements after processing the recorded spectra was the finding of the association constant and the stoichiometry of the complex.

### 3.3. Isothermal Titration Calorimetry

Isothermal titration calorimetry (ITC) measures the heat exchange during the complexation process, in this case, DHTH and β-cyclodextrin, being the key method to describe the interaction mechanisms between cyclodextrin and the included molecules [59].

The thermodynamic parameters characterizing their interactions were determined through isothermal calorimetric titration conducted at 25 °C using a Nano ITC 2G device (TA Instruments, New Castle, DE, USA).

In the isothermal titration calorimetry (ITC) experiment, a β-CD solution (0.75 mM) in the cell was stirred at 250 rpm, while a syringe was used to add the titrant solution of DHTH (3 mM) in a stepwise manner. Titration began once baseline stability was achieved, consisting of 25 consecutive injections of the ligand solution at 300 s intervals. Upon DHTH injection, molecular interactions generated heat, directly proportional to the amount of bound compound in each aliquot. A control experiment, where DHTH was titrated into water, provided a reference signal that was subtracted from the experimental data. The corrected heat data were analyzed using the independent binding model in NanoAnalyze 3.1.2 (TA Instruments, New Castle, DE, USA) software, yielding key thermodynamic parameters: the binding constant (K), stoichiometry (n), enthalpy change (ΔH), and entropy change (ΔS).

### 3.4. Molecular Docking

**Figure 16:**
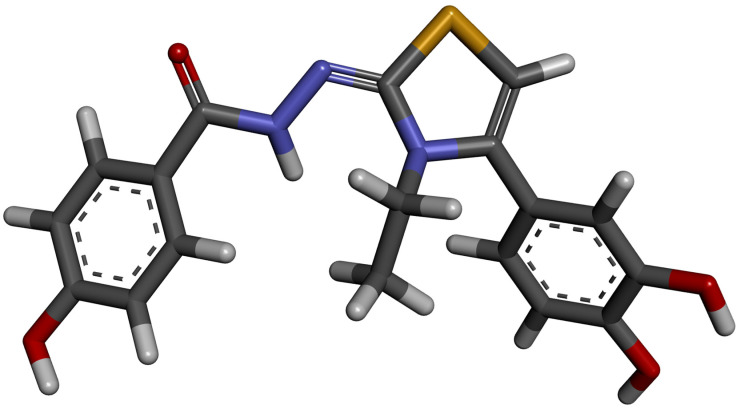
Optimized molecular structure of DHTH obtained by DFT.

Computational molecular docking was performed to obtain at molecular scale the most probable inclusion complex of DHTH inside β-cyclodextrin using AutoDock v4.2 [60]. For the molecular docking study, the tri-dimensional molecular structure of DHTH was optimized with Gaussian 16 software [61] using the meta-GGA M06-2X hybrid functional and the 6-311++G(d,p) basis set. No imaginary frequencies were obtained, which proves that this conformation corresponds to a minimum on the potential energy surface (PES) and not to a saddle point. The optimized structure of DHTH is presented in Figure 16.

The needed files as input were prepared using AutoDockTools 1.5.6 [60,62], following the AutoDock standard procedure, regarding the hydrogen atoms, the charges of which were calculated using the Gasteiger–Marsili method and the flexibility of DHTH, for which seven torsion angles were considered around the rotatable bonds [63]. Prior to the actual docking run, AutoGrid was used to precalculate grid maps with the size of the cube sides set to 60 points with 0.375 Å spacing, and the cubic search box was set relative to the β-CD molecule to surround it. The β-cyclodextrin 3D molecular structure was extracted from the crystallographic entry WERGUW from The Cambridge Structural Database, as reported by Caira et al. [64]. The Lamarckian genetic algorithm [65,66], which generates and optimizes iteratively a population of ligand conformations, was used to search for the best conformers, mainly sampling the global search space. The default parameters, automatically setup by AutoDockTools, were specified in detail in our previous contributions [51,67]. For good statistics and clustering, 2000 runs were performed, each of them launching with a random generation seed. Visual analysis of the docking results was performed using Biovia Discovery Studio Visualizer v20.1 and Chimera v1.14 [68].

### 3.5. UV Spectroscopy

A series of 20 mixtures comprising 1000 µL of DHTH in water (0.1 mM), 0–200 µL of β-CD in water (10 mM) and water to a total volume of 2000 µL were made and left for 1 h in the dark at room temperature on a rotary shaker. The UV spectra of the mixtures was recorded between 190 and 400 nm in 10 mm width quartz cuvettes on a Specord 210 PLUS UV-Vis (Analytik Jena AG, Jena, Germany).

### 3.6. Antioxidant Activity Evaluation

To assess the impact of β-CD incorporation on the antioxidant activity of DHTH, an in vitro α,α-diphenyl-β-picrylhydrazyl (DPPH) scavenging assay was conducted on both free and β-CD-complexed DHTH [3,69,70]. The solid DPPH was dissolved in ethanol until the constant absorption of the reagent solution was approximately A = 0.7 at 517 nm. An amount of 2 mL of the DPPH reagent solution was mixed with 500 µL of DHTH solution (0.1 mM) and a 500 µL solution of 0.1 mM DHTH and 0.2 mM β-CD. The absorption of the samples was measured every second for 5 min against a blank solution at room temperature.

## 4. Conclusions

A new non-colored polyphenolic antioxidant thiazole was successfully obtained and characterized. In the current study, the binding of DHTH to β-CD was analyzed using NMR spectroscopy, ITC calorimetry, UV spectroscopy, and molecular docking, which systematically extended the understanding of the interactions from the drug–β-CD binding process.

The ^1^H NMR spectroscopy indicated a moderate interaction between DHTH and β-CD. From the analysis of the 2D ROESY NMR spectrum, interactions were observed between aromatic and thiazole protons belonging to DHTH molecules and the cavity of β-CD, which proved the inclusion of DHTH inside β-CD. This confirms the formation of the DHTH:β-CD complex and predicts the geometry in the liquid state.

Thermodynamic data indicate a 1:1 guest–host stoichiometry, with the formation of the complex being driven by both enthalpic and entropic contributions, primarily due to hydrophobic interactions.

Two probable molecular conformations of DHTH were identified in the complex with β-CD, as resulted from the molecular docking calculations. The lowest theoretical binding energy ΔG = −6.32 kcal/mol was the most probable one identified, and an alternative pose with a binding energy ΔG = −5.23 kcal/mol was also identified.

The DPPH scavenging assay of the DHTH complexed in β-CD occurred with a reduced speed when compared to free DHTH, indicating a delaying of the antioxidant activity of the studied compound.

## Figures and Tables

**Table 1:** The chemical shifts, multiplicity, coupling constants for DHTH molecule in free state.

Proton Position	Number of Protons	Multiplicity	Coupling Constant (Hz)	Chemical Shift in Free State (ppm)	Type
**a**	2	d	J_a-b_ = 8.79	6.97	aromatic
**b**	2	d	J_b-a_ = 8.79	7.79	aromatic
**c**	1	s	-	6.88	thiazole
**d**	1	d	J_d-f_ = 2.20	6.96	aromatic
**e**	1	d	J_e-f_ = 8.22	6.98	aromatic
**f**	1	dd	J_f-e_ = 8.22 J_f-d_ = 2.20	6.90	aromatic
**g**	3	t	J_g-h_ = 7.33	1.24	methyl
**h**	2	q	J_h-g_ = 7.33	4.00	methylene

## Data Availability

Data are contained within the article or Supplementary Materials.

## Supplementary Materials

The following supporting information can be downloaded at: https://www.mdpi.com/article/10.3390/molecules30081842/s1, Figure S1: The mass spectrum recorded for compound **3**–negative ionization; Figure S2: The mass spectrum recorded for compound **5** (DHTH)–negative ionization; Figure S3: The infrared spectrum recorded for compound **3**; Figure S4: The infrared spectrum recorded for compound **5** (DHTH); Figure S5: The 1H NMR spectrum recorded for compound **3**; Figure S6: The 1H NMR spectrum recorded for compound **3**; Figure S7: The 13C NMR spectrum recorded for compound **5** (DHTH).
