# Supplementary material for: New Antioxidant Triphenol-Derived Hydrazide-Hydrazone Thiazole: Formation and Analysis of Inclusion Complex with β-CD Using Experimental and Computational Approaches
Source: Molecules. 2025 Apr 19;30(8):1842. doi: 10.3390/molecules30081842 (PMC12029814; doi:10.3390/molecules30081842)
Supplement: Supplementary file 1 [file molecules-30-01842-s001.zip › molecules-3587235-supplementary.pdf]

molecules

New Antioxidant Triphenol-Derived Hydrazide-Hydrazone Thiazole: Formation and Analysis of Inclusion Complex with β-CD Using Experimental and Computational Approaches

Adrian Pîrnău [1], Mihaela Mic [1,*], Călin G. Floare [1], Ovidiu Oniga [2], Smaranda Dafina Oniga [3], Ovidiu Crișan [4], Laurian Vlase [5] and Gabriel Marc [4]

1. Figures

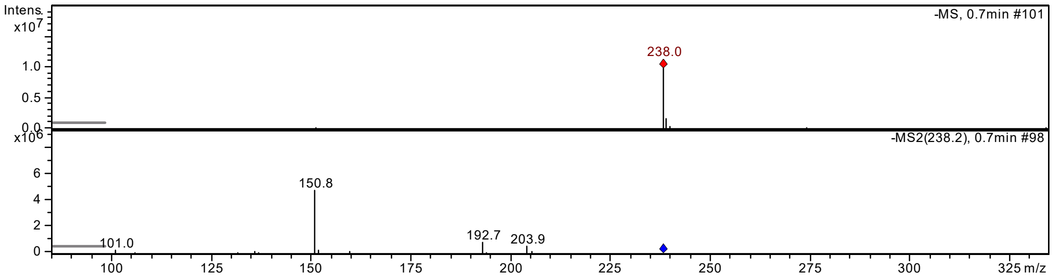

Figure S1. The mass spectrum recorded for compound **3** – negative ionization

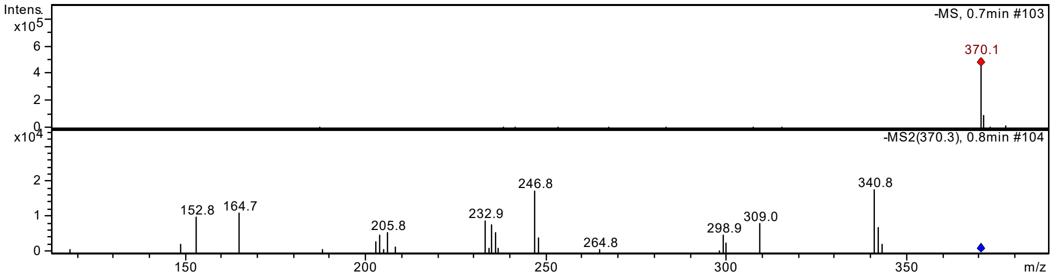

Figure S2. The mass spectrum recorded for compound **5 (DHTH)** – negative ionization

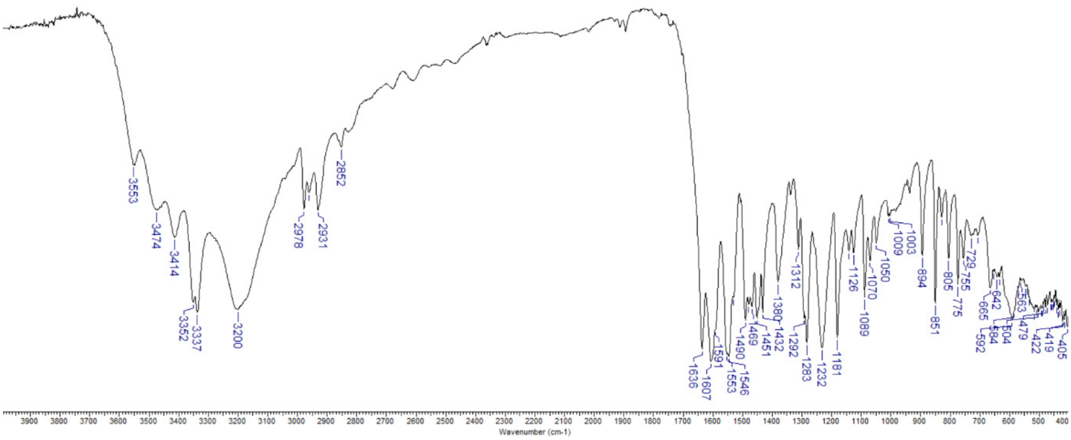

Figure S3. The infrared spectrum recorded for compound **3**

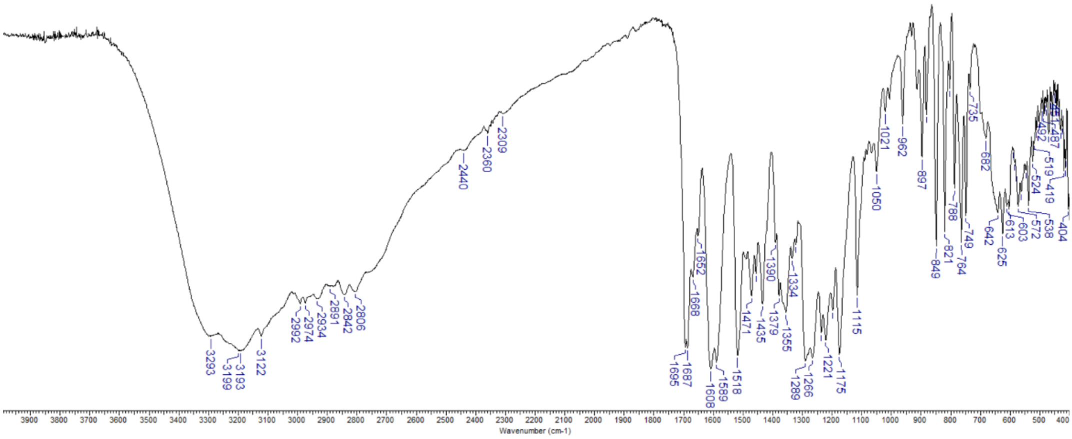

Figure S4. The infrared spectrum recorded for compound **5 (DHTH)**

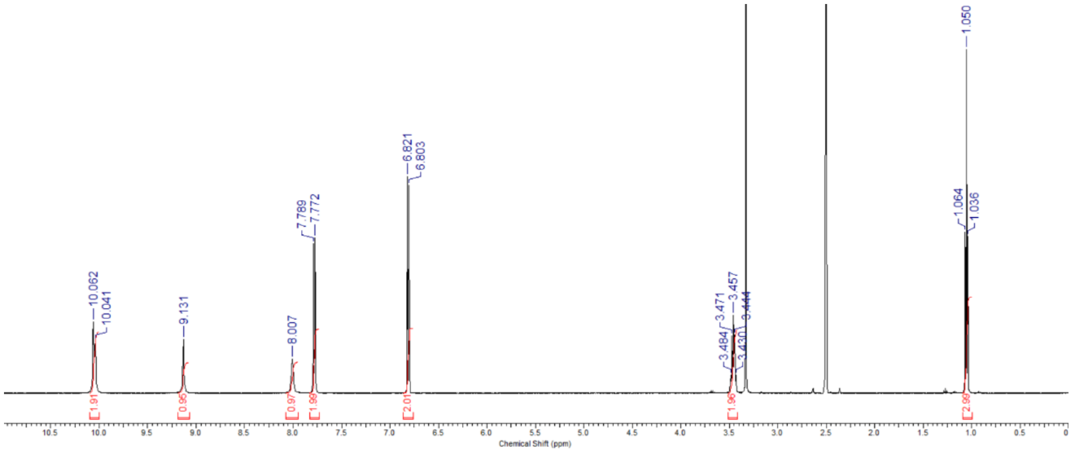

Figure S5. The 1H NMR spectrum recorded for compound **3**

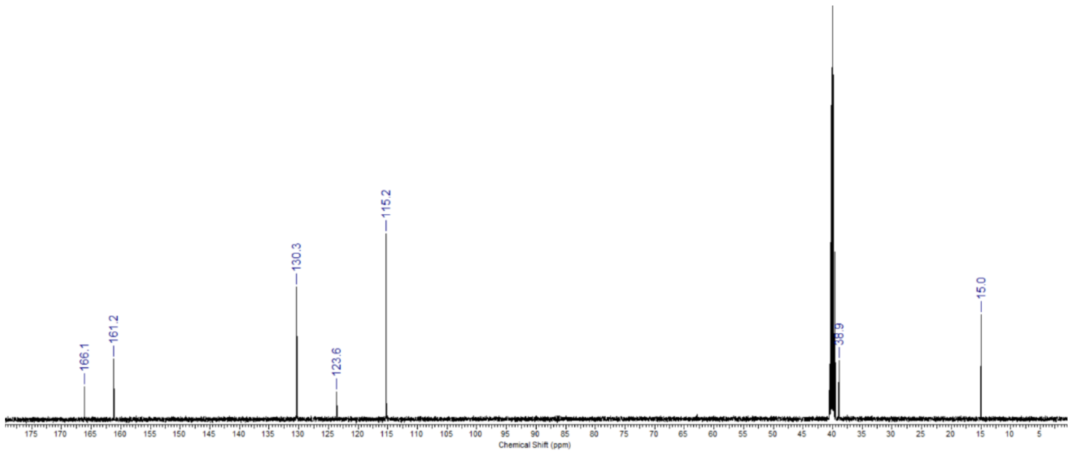

Figure S6. The 13C NMR spectrum recorded for compound **3**

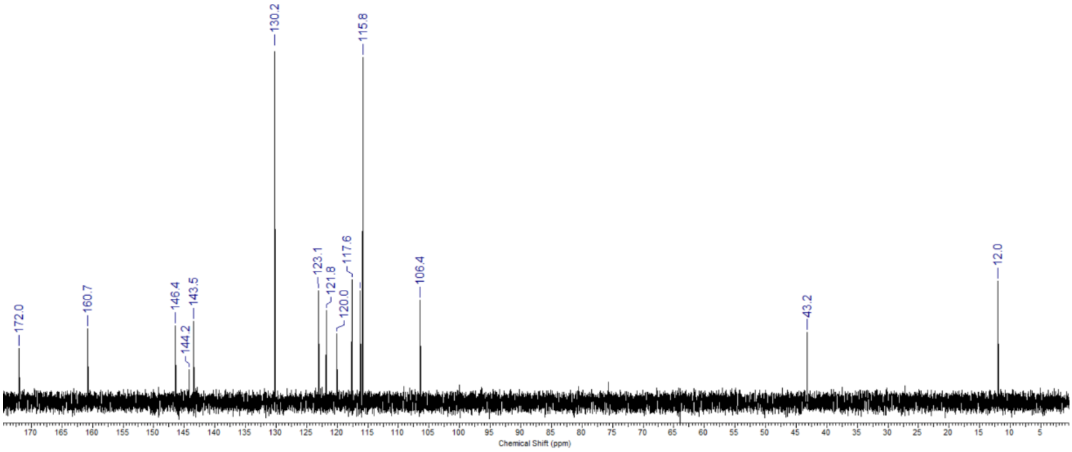

Figure S7. The 13C NMR spectrum recorded for compound **5 (DHTH)**
